# 3D Self-Supported Nitrogen-Doped Carbon Nanofiber Electrodes Incorporated Co/CoO_x_ Nanoparticles: Application to Dyes Degradation by Electro-Fenton-Based Process

**DOI:** 10.3390/nano11102686

**Published:** 2021-10-12

**Authors:** Ahmed Barhoum, Therese Favre, Syreina Sayegh, Fida Tanos, Emerson Coy, Igor Iatsunskyi, Antonio Razzouk, Marc Cretin, Mikhael Bechelany

**Affiliations:** 1 NanoStruc Research Group, Chemistry Department, Faculty of Science, Helwan University, Cairo 11795, Egypt; 2 Institut Européen des Membranes (IEM), UMR 5635, Université Montpellier, École Nationale Supérieure de Chimie de Montpellier (ENSCM), Centre National de la Recherche Scientifique (CNRS), Place Eugène Bataillon, 34095 Montpellier, France; therese.favre@ensiacet.fr (T.F.); syreina.sayegh@gmail.com (S.S.); fidatanos@gmail.com (F.T.); marc.cretin@umontpellier.fr (M.C.); 3 School of Chemical Sciences, Fraunhofer Project Centre, Dublin City University, D09 V209 Dublin, Ireland; 4 Laboratoire d’Analyses Chimiques, Faculty of Sciences, LAC—Lebanese University, Jdeidet 90656, Lebanon; carlorazzouk@hotmail.com; 5 NanoBioMedical Centre, Adam Mickiewicz University, 3, Wszechnicy Piastowskiej Str., 61-614 Poznan, Poland; coyeme@amu.edu.pl (E.C.); igoyat@amu.edu.pl (I.I.)

**Keywords:** electro-Fenton process, organic pollutants, azo dyes, wastewater treatment, carbon nanofibers, self-supported electrodes, electrospinning

## Abstract

We developed free-standing nitrogen-doped carbon nanofiber (CNF) electrodes incorporating Co/CoO_x_ nanoparticles (NPs) as a new cathode material for removing Acid Orange 7 (AO7; a dye for wool) from wastewater by the heterogeneous electro-Fenton reaction. We produced the free-standing N-doped CNF electrodes by electrospinning a polyacrylonitrile (PAN) and cobalt acetate solution followed by thermal carbonation of the cobalt acetate/PAN nanofibers under a nitrogen atmosphere. We then investigated electro-Fenton-based removal of AO7 from wastewater with the free-standing N-doped-CNFs-Co/CoO_x_ electrodes, in the presence or not of Fe^2+^ ions as a co-catalyst. The electrochemical analysis showed the high stability of the prepared N-doped-CNF-Co/CoO_x_ electrodes in electrochemical oxidation experiments with excellent degradation of AO7 (20 mM) at acidic to near neutral pH values (3 and 6). Electro-Fenton oxidation at 10 mA/cm^2^ direct current for 40 min using the N-doped-CNF-Co/CoO_x_ electrodes loaded with 25 wt% of Co/CoO_x_ NPs led to complete AO7 solution decolorization with total organic carbon (TOC) removal values of 92.4% at pH 3 and 93.3% at pH 6. The newly developed N-doped-CNF-Co/CoO_x_ electrodes are an effective alternative technique for wastewater pre-treatment before the biological treatment.

## 1. Introduction

Water pollution continues to be one of the greatest challenges worldwide. To date, many different water pollutant types (i.e., inorganic, organic, and biological) have been described [1,2,3]. Organic pollutants, such as fertilizers, herbicides, pesticides, dyes, and drugs, are the source of many environmental problems [4]. Therefore, eco-friendly technology is crucially needed to transform organic pollutants present in the water into less toxic molecules or to completely degrade them into CO_2_ and H_2_O [5]. Traditional approaches, such as coagulation, adsorption, chemical oxidation, chemical reduction [6], photocatalytic oxidation [7], and enzymatic degradation [8], are widely exploited to eliminate organic pollutants from effluents. However, they display some limitations [9], such as the need for complementary treatments before their elimination at wastewater treatment plants. Some of these methods are not fully efficient and/or too expensive [10]. Fenton reaction has been largely investigated. Fenton reaction is characterized by a high efficiency, low cost, and relatively easy operation and maintenance for the degradation (oxidation) of many hazardous organic pollutants (e.g., dyes, drugs, herbicides, pesticides, fertilizers) [11,12]; its implementation is hampered by the need of storing and shipping concentrated H_2_O_2(aq)_ and by the generation of Fe^3+^ sludge as a by-product of the electrochemical oxidation process [10].

Fe^2+^_(aq)_ + H_2_O_2(aq)_ + H^+^_(aq)_ → Fe^3+^_(aq)_ + ·OH_(aq)_ + H_2_O (1)

O_2 (g)_ + 2H^+^_(aq)_ + 2e^−^ → H_2_O_2_ (2)

Fe^3+^_(aq)_ + e^−^ → Fe^2+^_(aq)_ (3)

Electrochemical approaches (e.g., electrocoagulation, electrosorption, and electrochemical oxidation or reduction) have attracted great attention for organic pollutants treatment [1]. These methods represent a versatile, efficient, cost-effective technology for many industrial wastewater systems. Organic pollutants in wastewater can be electrochemically degraded (oxidation) using: (i) direct electrochemical oxidation (DEO), in which electrons are directly transferred from the electrode surface without participation of other species or reagents; and (ii) indirect electrochemical oxidation (IEO), in which organic pollutants are oxidized using electroactive species (·OH radicals), generated in solution or at the electrode surface [3,13].

Electro-Fenton oxidation is an IEO process in which organic pollutants in wastewater are degraded by ·OH radicals generated by the Fenton reaction (Equation (1)) between electro-Fenton reagents (e.g., Fe^2+^ ions) and H_2_O_2_. Such H_2_O_2_ molecules are produced by electrochemical reduction in dissolved O_2_ (Equation (2)), supplied by the continuous bubbling of O_2_ gas at the cathode. Therefore, the electro-Fenton oxidation efficiency can be influenced by the cathode materials (e.g., composition, morphology, crystalline structure, specific surface area, pore size), electrolytes (e.g., electro-Fenton reagent, temperature, and pH), reaction time, and applied voltage influence [14]. At the cathode, the electro-Fenton reagent (Fe^2+^ ions) required for the electro-Fenton reaction (Equation (1)) is continuously produced through Fe^3+^ reduction (Equation (3)). H_2_O_2_ and Fe^2+^ then allow the production of ·OH that can oxidize (degrade) the organic pollutants in wastewater.

Graphite electrodes have been used for electro-Fenton oxidation of wastewater pollutants because of their availability, high electrochemical activity, and low cost [15,16,17]. However, O_2_ (gas) poor solubility in aqueous solutions (wastewater) limits the mass transfer reaction, causing a low oxidation efficiency. Therefore, many groups have tried to develop new cathode materials that include additional electrochemically active sites with transition metal (e.g., Fe-Cu [18], Pd-Pt [19], Pt-Au [19]), metal oxides (e.g., ZnO [20], Fe_2_O_3_ [21], TiO_2_ [22], CuO [23]), and hydroxide (Fe-Co-(OH)_x_) [11] nanoparticles (NPs) embedded in carbon matrixes. Metal/metal oxide NPs on the carbon electrode surface can be used as heterogeneous catalysts for the local generation of H_2_O_2_ during the electro-Fenton oxidation process. Graphite [24], graphenes [25], carbon nanotubes [26], carbon nanofibers [27], carbon sponges [28], and carbon felts [14] are among the electrode materials with good conductivity investigated for the electrochemical production of H_2_O_2_. On the other hand, Fe^3+^ sludge formation, due to the production of Fe^3+^, and the need for acidic pH (pH 2–4) for the electro-Fenton process can be solved by the mobilization of metal NPs (electrocatalyst) to overcome the narrow pH range required for the homogeneous electro-Fenton oxidation. Many groups have then investigated heterogeneous electro-Fenton-based oxidation at almost neutral pH without adding electro-Fenton reagents (Fe^2+^ ions) in the solution. The interest to develop such materials for the heterogeneous electro-Fenton process has been recently highlighted by the description of Co and/or Fe layered double hydroxyls modified carbon felts [11,12] and zeolite modified carbon felts [29,30]. As the electrochemical stability of these electrode materials was not optimal, we decided to test new electrode materials.

Carbon nanofibers (CNFs) are an attractive class of electrode material showing good adsorption, electrical conductivity, and catalysis [31,32]. CNFs exhibit great potentials in the fields of electrocatalysis [33], batteries [34], supercapacitors [35], fuel cells [35], biosensors [36,37,38,39], and biomedical applications [40]. CNF hydrophobic properties, fiber network structure, and large surface area to volume ratio explain their high adsorption/absorption capacity for organic pollutants [32]. Moreover, due to their excellent electrical conductivity and mechanical integrity, free-standing CNFs are attractive as stable free-standing electrodes for H_2_O_2_ electro-generation. However, the electrocatalytic performance of free-standing CNF electrodes is far from satisfactory and could be increased by modulating the fiber morphology and pore structure and by incorporating electrocatalyst NPs [41]. Recent studies have described the fabrication of CNF electrodes decorated by metal, metal oxide, and metal hydroxide NPs using solution electrospinning, followed by post-peroxidation in air and thermal carbonization under an inert atmosphere [42,43,44]. However, it is easier to synthesize in situ the electrocatalyst NPs within the CNFs. Moreover, this approach requires less chemicals and is more effective compared with the post-synthesis of electrocatalyst NPs using for instance wet chemistry techniques [45,46,47,48]. Furthermore, when using post-synthesis approaches, the weak interaction between electrocatalyst NPs and CNF matrix cannot prevent the electrocatalyst NPs growth/aggregation into larger particles, thus losing their size-dependent electrocatalytic activity.

Dyes are organic pollutants that are commonly used by many water-based industries for paper [49], pesticides [50], fertilizers [51], printing inks [52], textile finishing [53], and drug manufacturing. For instance, azo dyes represent more than 60–70% of all dye classes used in textile industries [54]. Azo dyes are harmful compounds that produce hazardous by-products through hydrolysis and oxidation reactions taking place in wastewater [55]. In this study, an efficient free-standing N-doped CNF electrode loaded with Co/CoO_x_ NPs for AO7 removal is described. The N-doped-CNF-Co/CoO_x_ electrodes have several advantages, including large surface area and low cost, low weight, easy handling, high chemical resistance, high thermal stability, and good conductivity. Moreover, they can combine adsorption and electrochemical indirect degradation of hazardous organic compounds either through the production of H_2_O_2_ from oxygen reduction (Equation (2)) or the electro-Fenton process in presence of a catalyst (Equation (1)). Innovation in the present work involves the in situ synthesis of the electrocatalyst Co/CoO_x_ NPs into free-standing N-doped-CNF electrodes. The N-doped-CNF-Co/CoO_x_ electrodes were fabricated using an attractive route that combines electrospinning of the PAN/cobalt acetate solution, thermal peroxidation under air, and thermal carbonation under nitrogen atmosphere. The structural and electrochemical characterization of the N-doped-CNF-Co/CoO_x_ electrodes revealed that they were very stable at almost neutral pH and presented an excellent AO7 mineralization rate at different pH values (pH 3 and pH 6). The correlation between performance and electrode properties (composition, crystal structure, and morphology) was investigated. N-doping of CNFs was inspired by our work on N-doped graphitized carbon electrodes for the removal of bio-refractory compounds [56]. N-doping leads to the reduction in O_2_ adsorption/adsorption energy at the electrode surface and an improvement in the electrocatalytic properties of the surface toward oxygen reduction reaction [57,58,59].

## 2. Experimental 

### 2.1. Materials

N-doped-CNF-Co/CoO_x_ electrodes were fabricated using PAN (Mwt ~150,000, Sigma-Aldrich, St Quentin Fallavier, France), cobalt (II) acetate tetrahydrate (Co(OCOCH_3_)_2_·4H_2_O, 99.9%, Sigma-Aldrich), N,N-dimethylformamide (DMF, 98%, Sigma-Aldrich), and ethanol (C_2_H_5_OH, 99%, Sigma-Aldrich). The AO7 dye was used as a model organic pollutant for degradation experiments, without any additional purification step. The AO7 solution used in the experiments was prepared with sulfuric acid (H_2_SO_4_, 97%, Sigma-Aldrich), sodium hydroxide (NaOH, 98%, Sigma-Aldrich), iron (II) sulfate heptahydrate (Fe_2_SO_4_.7H_2_O, 98%, Sigma-Aldrich), and anhydrous sodium sulfate (Na_2_SO_4_, 98%, Sigma-Aldrich) (all analytical grade) and deionized water.

### 2.2. Fabrication of Composite Carbon Nanofiber Electrodes

To fabricate free-standing N-doped-CNF electrodes decorated with Co/CoO_x_ NPs (CNF, CNF-Co_3_, CNF-Co_10_, and CNF-Co_20_), the cobalt (II) acetate/PAN solution was electrospun and then subject to peroxidation and thermal carbonation under a nitrogen atmosphere. Briefly, after addition of 2 g PAN to the cobalt (II) acetate solution, the mixture was mixed overnight until a homogeneous polymeric solution was formed. Four precursor solutions (samples labeled as: CNF, CNF-Co_3_, CNF-Co_10_, and CNF-Co_20_) with different cobalt acetate: PAN weight ratios (0%, 3%, 10%, and 20%) were prepared and transferred to 20 mL syringes. The solutions were then electrospun through hypodermic needles (21 G) on a drum collector covered with Al foil (tip-to-collector distance = 14 cm, applied voltage = 25 kV, and flow rate = 1 mL/min). The as-collected nanofibers were first stabilized (crosslinked) at 250 °C under air atmosphere for 2 h, followed by carbonization at 1000 °C for 1 h (heating/cooling rate = 2 °C·min^−1^ under N_2_ atmosphere) to obtain the N-doped-CNF-Co/CoO_x_ electrodes.

### 2.3. Characterization of Electrode Material

The crystal structures and crystallinity of the N-doped-CNF and N-doped-CNF-Co/CoO_x_ electrodes were investigated using X-ray powder diffraction (XRD, PANalytical Xpert-PRO diffractometer with an accelerator detector using Cu-radiation and Ni-filtered with a wavelength of 0.154 nm) [60]. The electrode morphology, microstructure, and size distribution were studied using scanning electron microscopy (SEM, Hitachi S4800, Japan) and high-resolution transmission electron microscopy (HR-TEM, JEOL ARM200F). Element composition mapping was determined by energy-dispersive X-ray (EDX) spectroscopy analysis using a Zeiss EVO HD15 microscope equipped with an Oxford X-MaxN EDX detector and the HRTEM-JEOL ARM200F working at 200 eV [61]. The N-doped-CNF-Co/CoO_x_ electrode surface composition and binding energy were determined using X-ray photoelectron spectroscopy (XPS, Escalab 250, Thermo Fisher Scientific, Dardilly, France). The N-doped-CNF-Co/CoO_x_ electrode cobalt content was assessed by atomic absorption spectroscopy (AAnalyst 400, PerkinElmer, Villebon sur Yvette, France). To obtain Raman spectra, a LabRam ARAMIS spectrometer (HORIBA Xplora, Montpellier, France) was used with a diode laser delivering an exciting line at 659 nm. The spectrometer (laser beam) was focused on a 50× objective and diffused light of 1800 lines·mm^−1^ grating with acquisition at a continuous mode of 7 s, snapshot time of 15 s, and with a number of the accumulation set to 4 times. The thermal stability and ash contents in the prepared electrodes were determined by thermal gravimetirical anaylsis (TGA, TA Instrument, Q5000 SA, Guyancourt Yvelines, France). The N-doped-CNF-Co/CoO_x_ electrode magnetic behavior was characterized using a vibrating-sample magnetometer (VSM) (MPMS-XL SQUID VSM, Les Ulis, France) with a 7 T magnet and a magnetic field ranging from −50 to 50 kOe and a dc magnetization option. The specific surface areas were calculated using the Micrometric ASAP 2010 system and the Brunauer–Emmett–Teller (BET) method (outgassing conditions; 200 °C for 12 h). The pore size (diameter and volume) of the prepared electrodes was calculated with the Barrett, Joyner, and Halenda (BJH) method.

### 2.4. Electro-Oxidation of AO7

AO7 degradation and mineralization were tested using a two-electrode set-up in an open undivided cell (3 × 3 × 3 cm^3^) under continuous magnetic stirring and at constant current (10 mA·cm^−2^) supplied by a DC power supply. The cathodes were the free-standing N-doped-CNF and N-doped-CNF-Co/CoO_x_ electrodes, and the anode was a Pt mesh. Before electrolysis initiation, the cathode and anode were immersed (pre-saturated) in the AO7 solution under O_2_ bubbling for 15 min to prevent the experimental error of total organic carbon (TOC) decrease due to the AO7 molecule sorption onto the N-doped-CNF-Co/CoO_x_ electrodes. The AO7 solution was prepared from 0.1 mM AO7, 0.05 M Na_2_SO_4_ as a supporting electrolyte, and a temperature of 25 °C. The pH of the solution was adjusted by 0.1 M NaOH or 0.1 M H_2_SO_4_ solutions. The AO7 degradation (initial concentration of 0.1 mM) was compared in near neutral (pH 6, as raw pH) and acidic (pH 3 adjusted with H_2_SO_4_) aqueous solutions with 0.05 M Na_2_SO_4_ as supporting electrolyte and with/without Fe^2+^ as homogeneous electro-Fenton catalyst (0.2 mM from FeSO_4_·7H_2_O). AO7 concentration during electrolysis was determined with a U-3010 UV-vis spectrophotometer (Hitachi Co., Issy-les-Moulineaux, France) in samples (1.5 mL) collected from the reactor at different time points. After the measurement, samples were put back in the reactor to maintain the solution volume constant during the experiments. AO7 solution color change was determined by comparing the AO7 solution absorbance at 480 nm at baseline and after electrolysis. The AO7 solution TOC values at baseline and after electrolysis were measured with a TOC analyzer (Shimadzu, Noisiel, France).

## 3. Results and Discussion

The performance of electrospun CNFs with incorporated metal/metal oxide NPs for organic compounds removal by electro-Fenton oxidation has not been extensively studied [27]. Here, free-standing N-doped-CNF and N-doped-CNF-Co/CoO_x_ electrodes were fabricated and their use for electrochemical degradation of AO7 in an aqueous solution was tested. The production of N-doped-CNFs and N-doped-CNF-Co/CoO_x_ electrodes usually involves two steps: (i) thermal peroxidation (stabilization) of the as-prepared PAN or cobalt acetate/PAN nanofibers at 250 °C under air atmosphere; and (ii) thermal carbonization (pyrolysis) of the preoxidized nanofiber mat at 1000 °C under nitrogen atmosphere. Importantly, during the peroxidation step, PAN undergoes cyclization and partial dehydrogenation that make the polymer (PAN or cobalt acetate/PAN) fibers denser and more thermally stable during the subsequent thermal carbonization step. In this second step, the polymer (PAN or cobalt acetate/PAN) begins to pyrolyze with a considerable release of volatile by-products. As PAN is a polymer with nitrogen (N) atoms in its chain, it is used as N-source for CNF doping [62]. The diameter and morphology of the prepared CNF electrodes and their electrocatalytic activity were significantly influenced by the weight ratio of cobalt acetate to PAN. PAN thermal treatment under air (O_2_) led to the loss of two H atoms from adjacent C atoms in the main chain, and consequently, to the escape of H_2_O molecules and the formation of C=C double bonds. N atoms in the polymer (PAN) chain break their nitrile (-N=C-) bonds and form new bonds with other close C atoms, leading to a hexagonal cyclic structure. During the pyrolysis step (under N_2_ atmosphere), the cyclic structure opens, carbon chains are formed, and HCN escapes. The carbon chains form N-doped graphene nanosheets (graphitic structure with nitrogen defects), as previously described [62]. The roles of these N atoms in the electrocatalytic reactions have been discussed in the literature [63]. Recent studies on heat-treated Fe-based [64,65] and Co-based [66] N-containing catalysts showed that an increase in pyrrolic N and a decrease in pyridinic N can lead to enhanced ORR catalytic performance. The huge variation in the reported results is explained by differences in electrode fabrication methods, reaction precursors, electrochemical oxidation conditions, etc. [63,67]. In the next sections, the composition, crystal structure, and morphology of free-standing N-doped-CNF and N-doped-CNF-Co/CoO_x_ electrode materials will be investigated using the XRD, TGA, SEM, HR-TEM, XPS, Raman spectroscopy, and VSM and BET methods. The electrochemical behavior of the electrodes, including the electrochemical activity towards AO7 degradation, will be disused in detail.

### 3.1. Material Composition and Morphology

**Figure 1:**
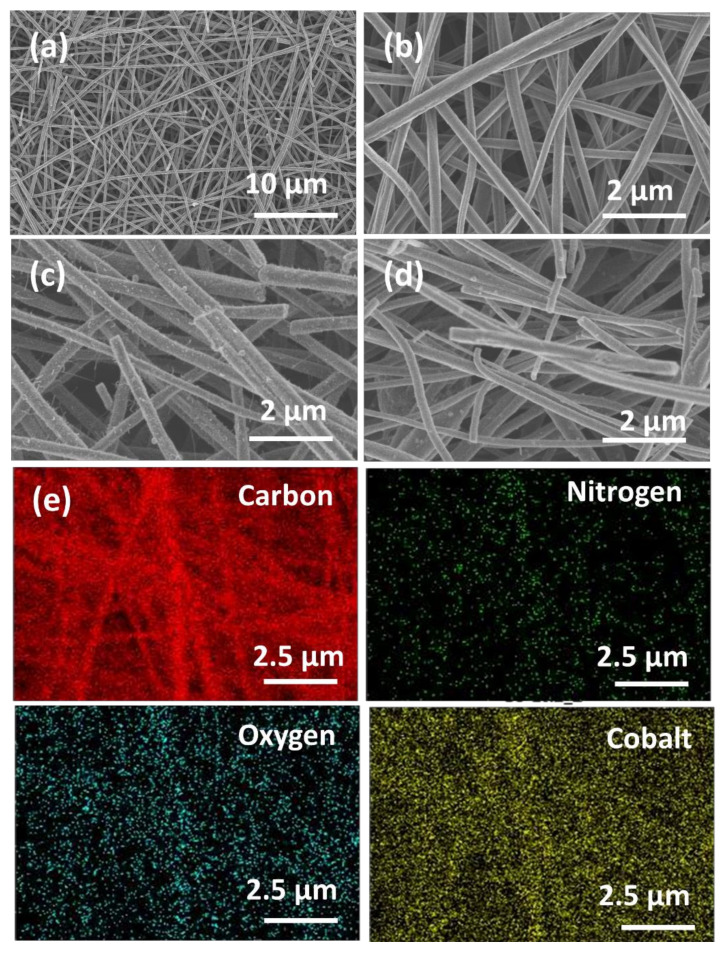
Field emission scanning electron microscopy images (FESEM) of the indicated CNF electrodes: (**a**) CNF; (**b**) CNF-Co3; (**c**) CNF-Co10; (**d**) CNF-Co20; and (**e**) SEM-EDX elemental mapping of carbon, nitrogen, oxygen, and cobalt in CNF-Co10.

PAN thermal pyrolysis led to the formation of randomly distributed N-doped-CNFs with a 3D-network structure (representative scanning electron microscopy (SEM) image in Figure 1a). The N-doped-CNF fiber diameter increased from 300 nm to 400 nm upon the increase in the cobalt acetate/PAN weight ratio from 3% to 20% (Figure 1b–d). The elemental composition (C, O, N, Co) and their distribution were further studied by EDX element mapping of CNF-Co10 (Figure 1e). The good matching of the C, O, N, and Co edges with the 3D nanofiber network morphology indicated that these elements were homogeneously dispersed in the entire structure. This feature might contribute to excellent electrochemical activity. SEM-EDX with the elemental mapping of CNF-Co10 (Figure 1e) confirmed that the tested materials were composed of 65% carbon, 5% nitrogen, 9% oxygen, and 21% cobalt. N-doping of CNFs can produce defects (catalytic active sites) in graphitic CNF structures and increase the electroactivity of the material, especially toward oxygen reduction. Previous research indicated that electrocatalytic activity is higher in N-doped than undoped CNFs, forming a stronger interaction with ions in the electrolytic solution [31]. However, CNF graphitization degree is not influenced by N absence or presence during thermal pyrolysis (carbonization) at different temperatures [41].

**Figure 2:**
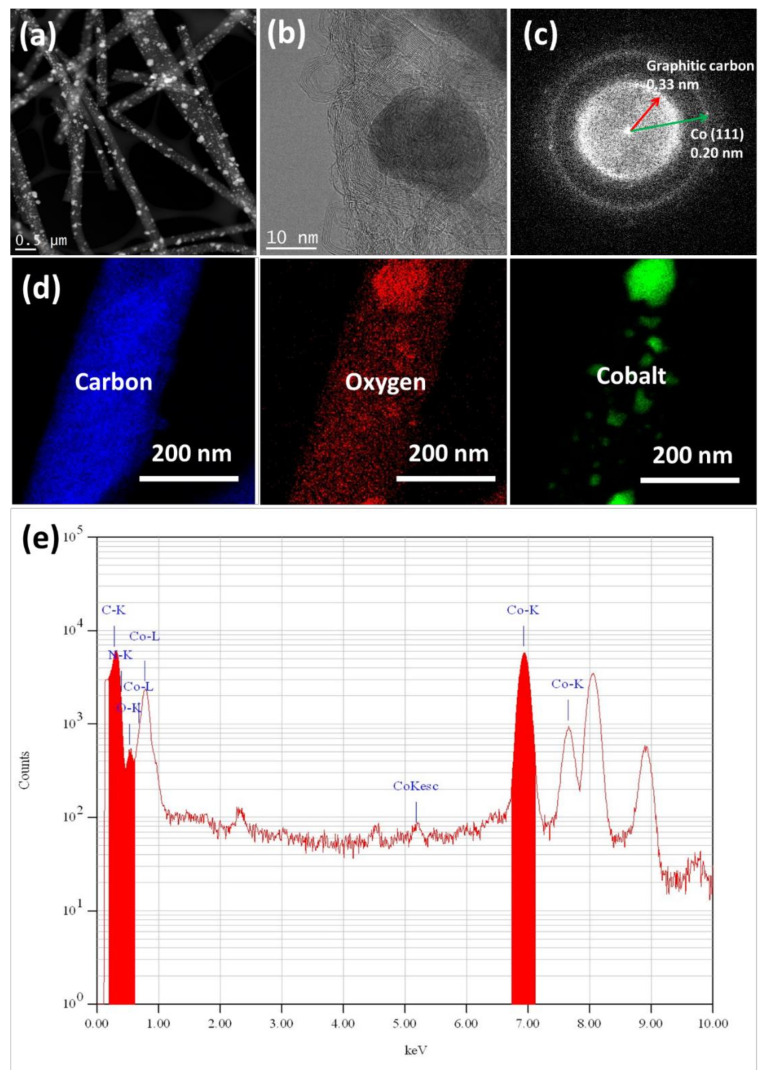
HR-TEM analysis of the CNF-Co20 electrode: (**a**) low magnification and (**b**) high magnification TEM micrographs; (**c**) SAED patterns; (**d**) TEM with elemental mapping; and (**e**) TEM-EDX spectra.

To further confirm the existence of Co/CoO_x_ NPs and their distribution in the CNF matrix, the CNF-Co20 electrode was analyzed by HR-TEM and selected area electron diffraction (SAED) patterns were generated. The Co/CoO_x_ NPs appeared as white particles that were homogeneously distributed on the CNF surface (TEM micrograph in Figure 2a). Moreover, high magnification TEM images (Figure 2b) showed that graphitic layers covered such Co/CoO_x_ NPs (~15 nm). This coating may protect NPs from dissolution during electrolysis in acidic conditions. The ambiguous lattice fringes of CNF-Co20 and the clear diffraction rings (Figure 2c) confirmed the presence of Co/CoO_x_ NPs and the formation of graphitic layers with a high crystalline structure on the CNF surface. Crystalline graphitic layers separated the Co/CoO_x_ NPs (d-spacing = 0.33 ± 0.02 nm). The electrocatalyst Co/CoO_x_ NPs showed a d-spacing of about 0.20 ± 0.02 nm that corresponded to the (1 1 1) plane of the face-cantered cubic (fcc) crystal structure of Co/CoO_x_ nanocrystals [68]. The TEM-EDX and TEM images with the elemental mapping of CNF-Co20 are shown in Figure 2d,e, respectively.

**Figure 3:**
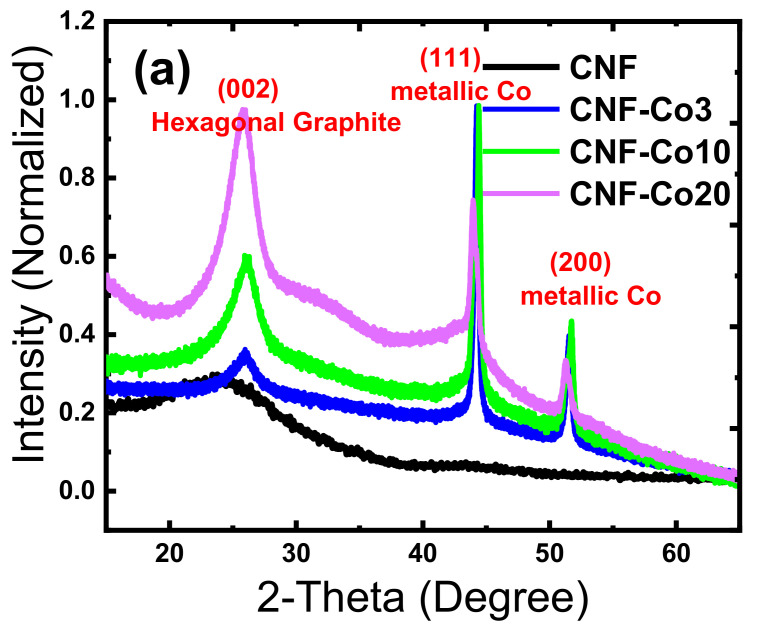

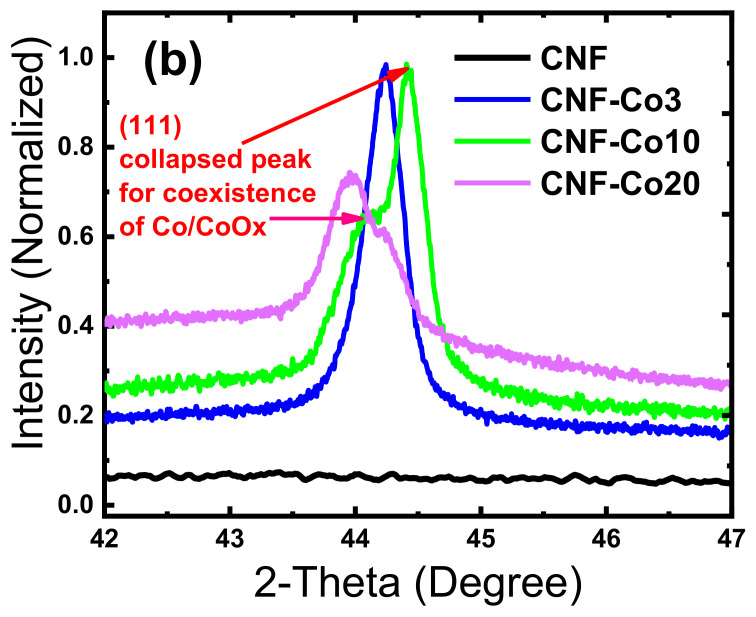
XRD profiles of the prepared free-standing electrodes: (**a**) survey XRD scan; (**b**) high-resolution scan of 111 peak(s) around 44°.

The XRD patterns of the N-doped-CNF and N-doped-CNF-Co/CoO_x_ materials are shown in Figure 3a. The Bragg reflection at 2θ = 25° corresponded to the (002) lattice planes of hexagonal graphite (JCPDS card no. 41-1487) with an inner-layer spacing d = 0.33 nm. This suggests that all as-obtained CNF samples possessed a low degree of graphitization [69]. However, compared with CNF-Co3, CNF-Co10, and CNF-Co20, the (002) peak intensity of CNFs was stronger and higher, suggesting a better crystallinity of the CNF matrix. CNF loaded with Co/CoO_x_ NPs exhibited three clear peaks at around 44.3° and 54° that are attributed mainly to the (111) and (200) planes of fcc crystal structures of metallic Co (JCPDS 15-0806), respectively. This indicated that the Co/CoO_x_ NPs were well distributed within the CNFs. The high-resolution scan of the (111) peak at 2θ of 44° showed two collapsed diffraction peaks only for CNF-Co10 and CNF-Co20, but not for CNF and CNF-Co3 (Figure 3b). These results confirmed the presence of a small amount of CoO_x_ that can be detected by XRD. As cobalt acetate is a thermally unstable compound, the high cobalt acetate concentration used in the preparation of CNF-Co10 and CNF-Co20 led to (i) an increase in the amount of cobalt acetate in direct contact with air during the peroxidation step; (ii) an increase in the degradation and oxidation of cobalt acetate on the cobalt acetate/PAN surface to form CoO_x_ clusters before pyrolysis of the cobalt acetate/PAN nanofibers under nitrogen atmosphere. Previous studies [63] reported that the existence of Co/CoO_x_ heterojunctions significantly enhances the electrocatalytic performance of the electrodes. The generation of metallic Co nanocrystals as a domain rather than CoO_x_ can be the result of the production of reducing by-products during thermal pyrolysis at lower pressure [70]. However, it is important to emphasize that the diffraction peaks of CNF-Co20 and CNF-Co10 appeared at smaller or larger 2-theta angles than that of CNF-Co3, respectively, indicating higher Co/CoO_x_ NPs content. Particularly, the increase in peak intensity at 25° and 44.3° indicates that Co/CoO_x_ NPs accelerate the formation of graphitic carbon.

**Figure 4:**
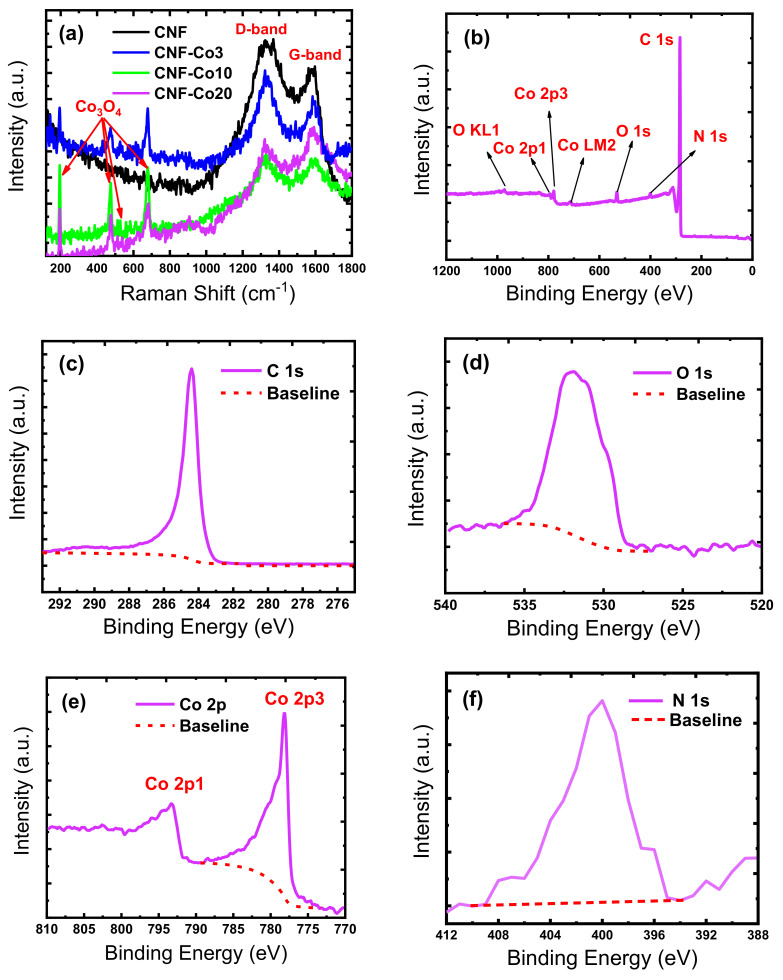
(**a**) Raman spectroscopy and XPS spectra of the prepared CNF-Co10 electrodes: (**b**) survey scan; (**c**) C 1 s; (**d**) O 1 s; (**e**) Co 2p; (**f**) N1s.

The co-existence of CoO_x_ and Co NPs and the influence of the cobalt acetate:PAN weight ratio on the degree of CNF graphitization were assessed by Raman spectroscopy (Figure 4a). For all tested electrodes, the typical D (disorder) peak at ~1342 cm^−1^ and the G (graphite) peak at ~1578cm^−1^ of the graphitic layers in CNFs [71] were observed. The D peak is not present in perfect graphite structures, because it belongs to the hybridized vibrational mode associated with the edges and graphitic defects. The G peak may be explained by the in-plane stretching vibration of all sp^2^-hybridized C-atom pairs in the graphitic structure of CNFs. The intensity ratio (R) of the D and G peaks (ID/IG) is often proportional to the CNF graphitization extent. The R-value decreases with higher CNF graphitization values [69]. Indeed, the R-value ranged from 1.2 (pure CNFs) to 1.1 (CNF-Co3), 0.9 (CNF-Co10), and 0.3 (CNF-Co20). This demonstrated that a graphite layer structure was progressively formed when the cobalt acetate/PAN weight ratio was increased from 3 to 20% [72]. Raman spectrometry also highlighted the presence of the three typical peaks for Co_3_O_4_ at 194.1, 471.8, and 667.4 cm^−1^. These peaks are associated with T_2_g, Eg, and A_1_g symmetries. Co_3_O_4_ presence is explained by cobalt acetate degradation and oxidation in contact with air during the peroxidation step [73].

The XPS spectra of the prepared electrodes were recorded to study the surface composition of the electrode graphite carbon nanostructures, their N-doping, and the formation of Co/CoO_x_ NPs heterojunctions. The survey scan of the CNF-Co20 electrode showed the differences in the chemical states of carbon (C 1s, ~284.45 eV), oxygen (O 1 s, ~531.5 eV), nitrogen (N 1 s, ~400.8 eV), and cobalt (Co 2p at ~778.4 eV). Analysis of the XPS spectra indicated that the C 1 s peak (Figure 4c) was explained by the existence of four carbon species with specific binding energy: C 1 s sp^2^ (C=C), sp^3^ carbon (C-C/C-H, 285.23 eV), (C-O, 285.88 eV), (C=O, 286.81 eV), and (C-O=C, 288.21 eV) [74]. The O 1 s peak (Figure 4d) could be decomposed in three peaks at 531.5 eV (O^2−^, corresponding to the oxide of cobalt II and/or III), and 532.2 eV (-OH, assigned to Co–OH groups). The peak at 536 eV was due to chemisorbed oxygen, and the peak at 533.6 eV to chemisorbed water. HR-XPS spectra of the Co 2p1/2 and Co 2p3/2 peaks (Figure 4f) confirmed the presence of CoO_x_ impurities. The CNF-Co20 electrode showed two satellites for the Co2p: Co 2p1/2 (770–790 eV) and Co 2p3/2 (centered at 778.4 eV) [75,76]. This indicated the formation of metallic Co NPs on the CNF surface with CoO_x_ as minor impurities, which is consistent with the SEM-EDX (Figure 1), TEM-EDX (Figure 2), XRD (Figure 3), and Raman spectra (Figure 4a). The N 1 s peak (Figure 4f) displayed oxidized N and three other N species (pyrrolic, pyridinic, and graphitic) [75] that interacted with three C atoms at different locations on the graphene layer (402–410 eV).

**Figure 5:**
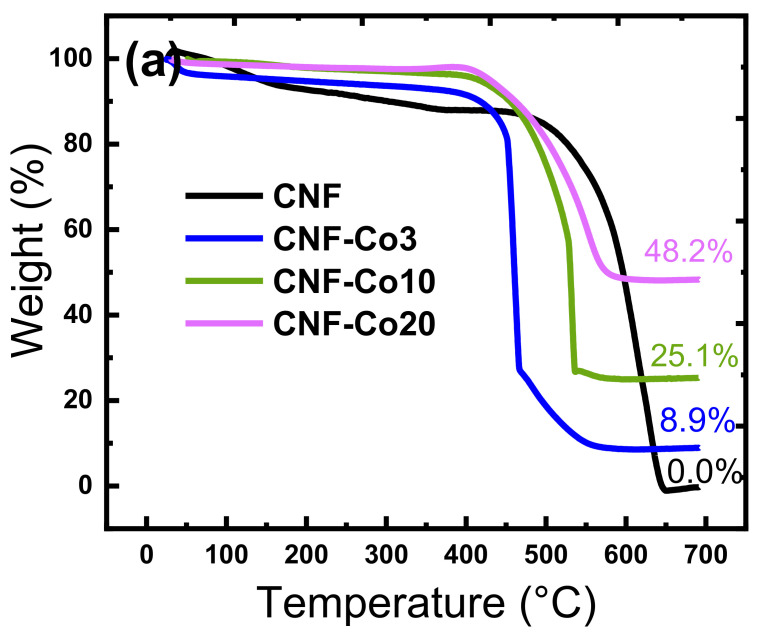

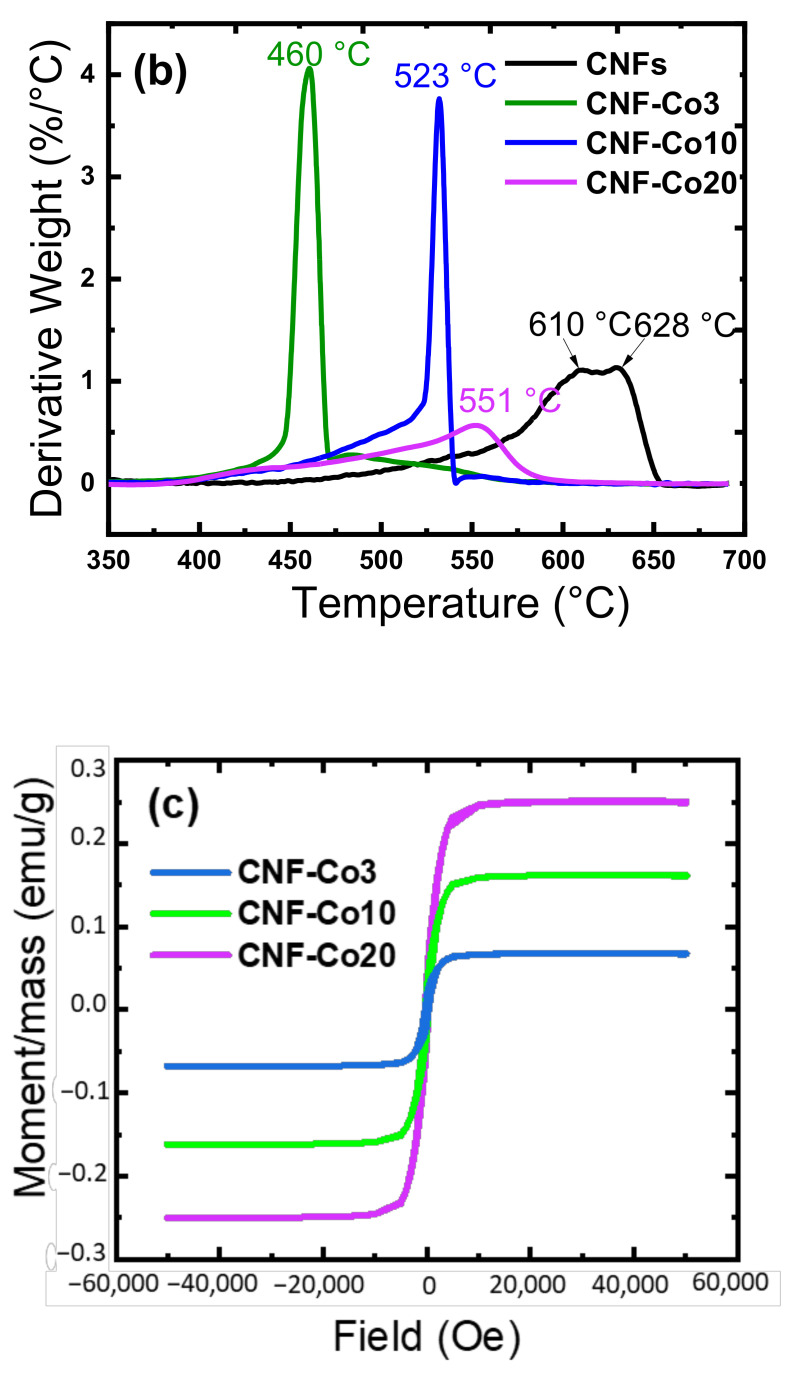
Features of the indicated electrodes: (**a**) TGA curves; (**b**) derivative TG curves; (**c**) VSM hysteresis curves for magnetism.

TGA of the free-standing N-doped-CNF and N-doped-CNF-Co/CoO_x_ electrodes was used to determine the effect of cobalt acetate incorporation on CNF thermal stability and ash (Co/CoO_x_) content (Figure 5 a,b). Analysis of the TGA curves of the CNF and CNF-Co/CoO_x_ materials in the air atmosphere showed a weight loss of ~4 wt% in all samples from 60 °C to 250 °C due to evaporation of the physically and chemically bound water and solvent (Figure 5). The N-doped-CNF-Co/CoO_x_ electrodes started to decompose at lower temperatures compared with the CNF electrode. This suggests that Co/CoO_x_ NPs behave as a catalyst for PAN oxidative degradation. Complete decomposition of pure CNF occurred in two steps at 610 °C and 628 °C. Conversely, oxidative degradation of CNFs loaded with Co/CoO_x_ NPs (CNF-Co3, CNF-Co10, CNF-Co20) occurred in one step at 460 °C, 523 °C, and 551 °C, respectively (Figure 5b). TGA analysis was used to determine the weight percentage of Co/CoO_x_ NPs relative to the CNFs. The residue at 700 °C attributed the Co/CoOx NPs content in the composite CNFs. The TGA results showed that the Co/CoO_x_ NPs content (residue) in the prepared samples were 8.9%, 25.1%, and 48.2% for CNF-Co3, CNF-Co10, and CNF-Co20, respectively.

Magnetic Co/CoO_x_ NPs have received considerable attention, because they offer unique advantages compared with other materials in terms of enhanced electrocatalytic applications. Therefore, CNF magnetization through Co/CoO_x_ NPs incorporation was studied by VSM [77]. The magnetic hysteresis loops of CNF-Co/CoO_x_ measured at room temperature (Figure 5c) showed that the saturation magnetization of CNF-Co3, CNF-Co10, and CNF-Co20 reached 0.067, 0.165, and 0.252 emu/g, respectively. The magnetization of N-doped-CNF-Co/CoO_x_ (emu/g) increased with the Co/CoOx content. Interestingly, the magnetic properties of Co/CoO_x_ NPs are increased by allowing the easy separation of any eluted Co/CoO_x_ NPs catalyst from the aqueous medium.

Many researchers have tried to increase the surface porosity and number of co-catalyst nanoparticles (catalytic sites) incorporated on/into the CNFs. To date, different systems of N-doped-CNFs incorporating metal/metal oxide NPs have been described [62]. Conversely, the effect of Co/CoO_x_ and N-doping on the electro-Fenton activity of the electrodes has not been investigated yet. The electrocatalytic performance of the electrode material is influenced by the number of catalytic sites (Co/CoO_x_ NPs), electrical conductivity (CNF diameter and graphitic structure), and CNF porous surface structure (BET surface area and pore diameter) [62].

The porous network structures (specific surface area and pore diameter) of electrodes significantly affect the electrochemical performance of the degradation of organic pollutants. It has been suggested that the pore interior dimensions (pore diameter) might restrict the possible orientations of a reactive molecule (organic pollutants). The BET surface area and pore size of free-standing N-doped-CNF and N-doped-CNF-Co/CoO_x_ electrodes were measured using nitrogen adsorption–desorption isotherms. The BET surface area values were 7.1 m^2^/g, 115.3 m^2^/g, and 240.2 m^2^/g, and the mean pore diameter values were 13.9 nm, 3.8 nm, and 5.6 nm for the CNF, CNF-Co10, and CNF-Co20 electrodes, respectively. According to the IUPAC nomenclature, mesoporous nanomaterials are materials with mean pore diameters between 2 nm and 50 nm. The high electrocatalytic activity of such nano-porous structures is attributed to high Co/CoO_x_ NPs dispersion among the graphitic mesoporous N-doped-CNF framework, which further accounts for the high electrocatalytic activity due to more “accessible” catalytic sites (Co/CoO_x_ NPs) for the reactants (ions, free radicals, and organic pollutants).

### 3.2. Application of Materials to the Electrochemical Degradation of the AO7 Dye

To understand the cathode composition impact on the degradation of AO7, CNF cathodes without/with immobilized Co NPs (CNFs, CNF-Co3, CNF-Co10, and CNF-Co20) and without/with the addition of 0.20 mM Fe^2+^ in solution (as Fenton catalyst) were used. All prepared cathodes presented the same solid surface area. Table 1 summarizes the electrode physical properties, the effective Co mass content (determined by TGA), the remaining concentration of AO7 (in %), determined by UV-vis spectroscopy (480 nm) at pH 3 after 40 min of electrolysis, the AO7 decolorization rate constant, and the TOC removal percentage at pH = 3 and pH = 6.

**Figure 6:**
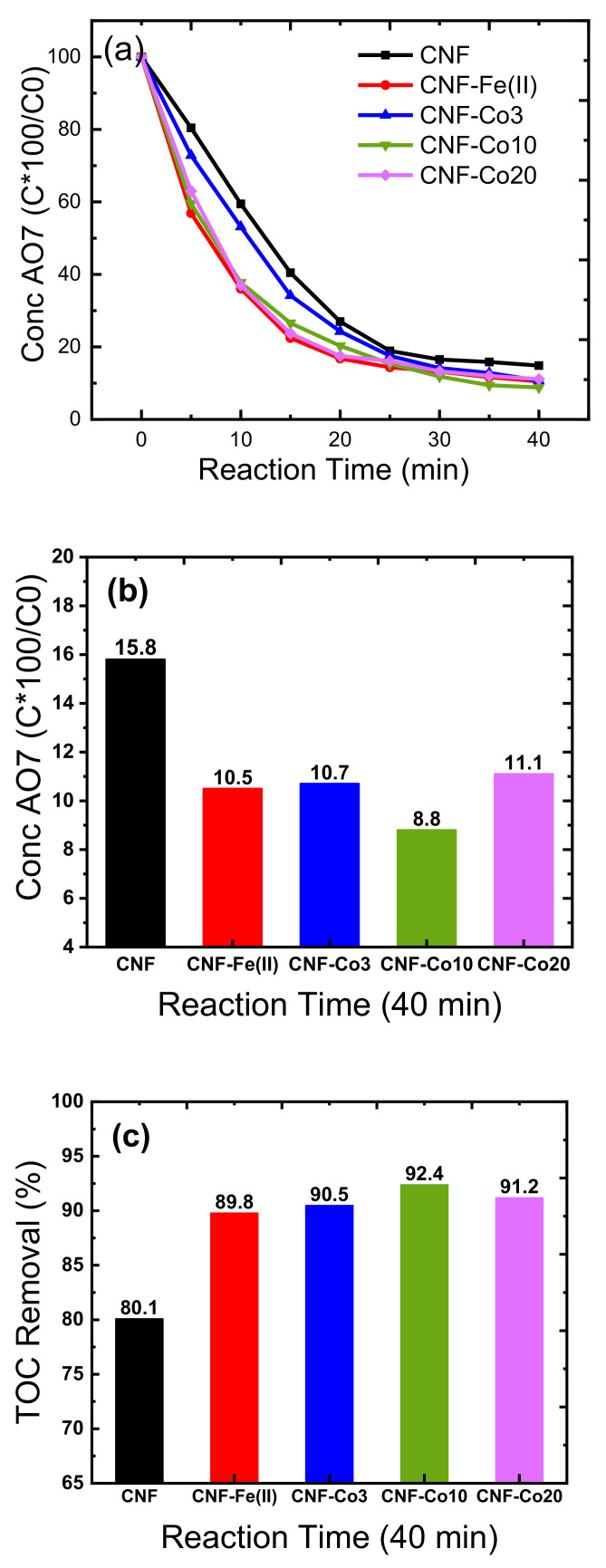

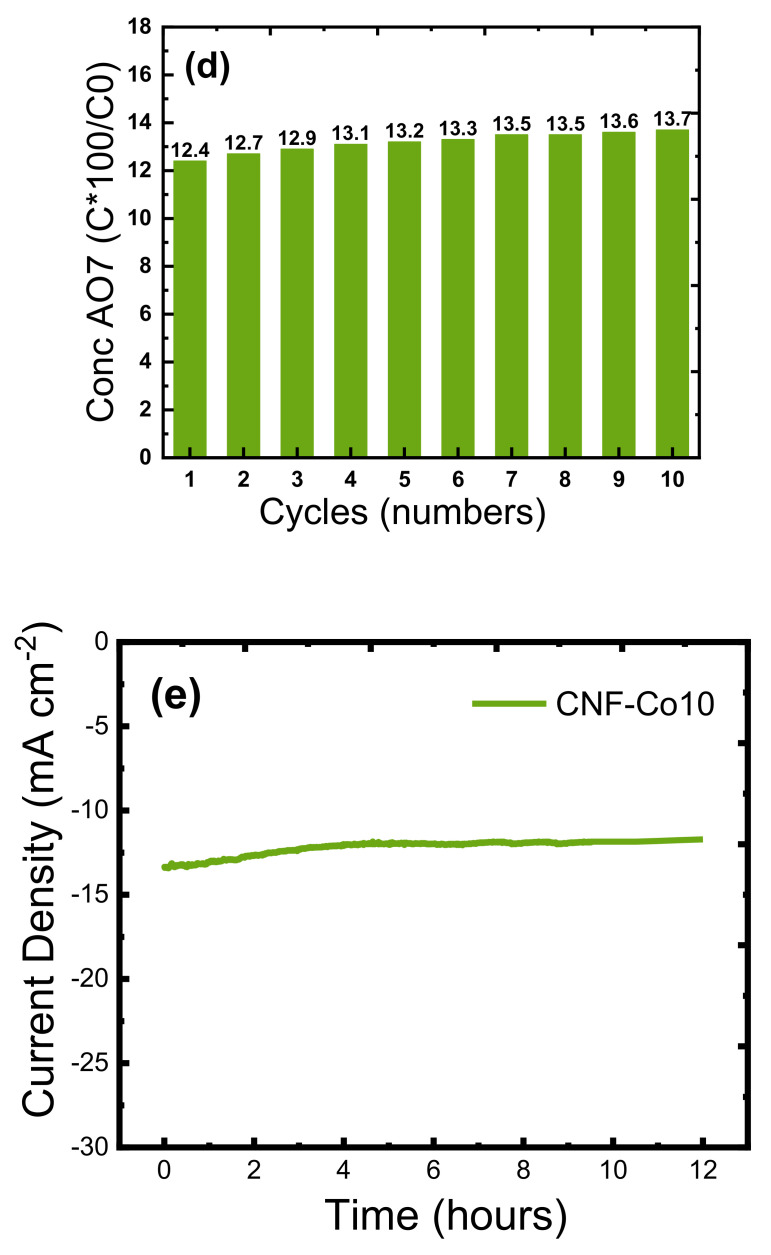

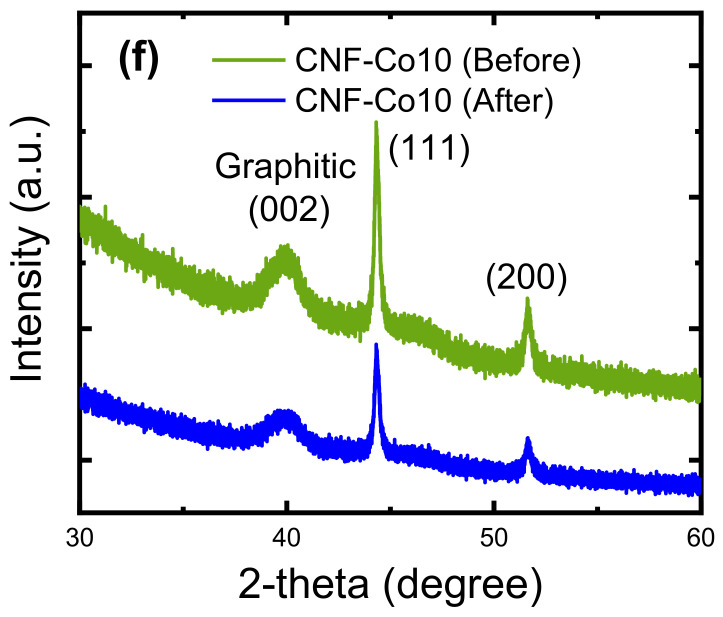
Influence of the cathode material on AO7 degradation (**a**) at different time points during the reaction and (**b**) after 40 min of electrolysis. (**c**) TOC removal (%) after 40 min of electrolysis. Conditions: applied current = 10 mA/cm^2^, electrolyte = 50 mM Na_2_SO_4_, initial concentration (C_0_) of AO7 = 0.1 mM, pH value = 3, reaction temperature = 25 °C. CNFs-Fe(II) is a pure CNF tested in the presence of 0.20 mM Fe^2+^ as electro-Fenton reagent. (**d**) Performance of the prepared CNF-Co10 electrode: after 10 cycles of electrolysis (40 min/cycle). Conditions: applied current = 10 mA/cm^2^, electrolyte = 50 mM Na_2_SO_4_, initial concentration (C_0_) of AO7 = 0.1 mM, pH = 6, reaction temperature = 25 °C; (**e**) XRD patterns of the CNF-Co10 electrode after 10 cycles of electrolysis (40 min/cycle); (**f**) the chronoamperometric response (j-t) test of the CNF-Co10 electrode in 0.5 M H_2_SO_4_ over 12 h.

A comparison of AO7 concentration over time (Figure 6a) clearly showed that the cathode material degradation performance was influenced by the Co NPs content. Indeed, the values of the apparent kinetic constants of AO7 degradation (based on a first-order kinetic) were 0.071 min^−1^, 0.080 min^−1^, and 0.089 min^−1^ for CNF-Co3, CNF-Co10, and CNF-Co20, respectively. Moreover, the apparent kinetic constant values were 0.068 min^−1^ and 0.090 min^−1^ when using the Co-free electrode without (CNF) and with Fe^2+^ in solution (CNFs-FeII). This confirms the performance of Co NPs as an electrocatalyst in heterogeneous electro-Fenton oxidation. For the CNF experimental condition without Fe^2+^ in solution, AO7 degradation might be due to its partial oxidation at the Pt anode and also to oxidation by H_2_O_2_ electrochemically produced at the CNF cathode from oxygen reduction. Altogether, these findings demonstrated the efficiency of cobalt NPs embedded in free-standing CNFs for the electrochemical activity of the materials. Moreover, a comparison of AO7 degradation after 40 min (Figure 6b) indicated that the optimal mass content of Co NPs in CNF (CNF-Co10) was about 25%. Moreover, it is well known that the optimal homogeneous electro-Fenton process occurs in solutions with a working pH value of 3. Indeed, the cathode oxidative capacity is weak if pH > 4 because of Fe (III) precipitation. Therefore, it is crucial to extend the electro-Fenton process to efficiently work at near-neutral pH values. Analysis of AO7 degradation at pH 6 after 10 cycles (40 min per cycle) using the CNF-Co10 electrode (with the optimal composition of N-doped-CNF-Co/CoO_x_) (Figure 6d) highlighted the electrode good stability and efficiency with a residual AO7 concentration of 12.4% after cycle 1, which is close to the value obtained at pH 3 (8.8%, see Figure 6b). This shows the efficiency of the as-prepared electrodes for the heterogeneous electro-Fenton process. The chemical stability and electrocataytic performance of the best CNF-Co10 electrode have been examined. Figure 6e shows the XRD patterns of the as prepared CNF-Co10 electrode and after 10 cycles of electrolysis (40 min/cycle); the XRD curves indicate that prepared electrodes are chemically stable over 10 cycles of electrolysis (40 min/cycle) of working time. Figure 6f shows the chronoamperometric response of CNFs electrodes remain stable over 12 h.

The results summarized in Table 1 suggest that CNFs-Co10 containing 25 wt% Co NPs is the best electrode for AO7 degradation by electro-Fenton oxidation. This reaction follows a pseudo-first-order reaction with the removal of 14–94% of AO7 in 1 h, in the function of the reaction conditions. After 40 min of electrolysis (10 mA/cm^2^ direct current), the CNFs-Co10 electrodes achieved complete AO7 decolorization with TOC removal of 92.4 ± 0.6 at pH 3 and 93.3 ± 0.5% at pH 6. The newly developed CNFs-Co10 electrodes are an effective alternative for wastewater pre-treatment before the biological treatment.

Then, the efficiency of the CNFs-Co10 electrodes was compared with that of cathodes described in some recent studies (Table 2). Le et al. [14] studied AO7 removal from wastewater by electron-Fenton oxidation with a graphene-based carbon felt cathode; AO7 (0.1 M) decolorization was observed in 5 min with AO7 mineralization (94%) after 8 h of electrolysis Le et al. [78] reported that the toxicity of 1 mM AO7 solution increases quickly during the first 5 min of electrolysis, when aromatic intermediates are formed, for instance, 1,4-benzoquinone and 1,2-naphthoquinone. Then, short-chain acids are formed, such as formic acid and acetic acid, before mineralization is completed at 270 min [78]. Lin et al. [79] evaluated the electrochemical oxidation (decolorization) of an AO7 aqueous solution using Fe_3_O_4_ activated peroxydisulfate (PDS). The aqueous AO7 solution was decolorized after 1 h of electrolysis with TOC removal of 20%. TOC removal increased to 30% after 90 min. The authors also reported that, during the first 30 min of the reaction, the main intermediate by-products were 1,4-benzoquinone and 1,2-naphthalenedione that are harmful because of the toxicity of quinones. However, after 30 to 90 min of reaction, these intermediates could be converted to less or non-toxic compounds [79]. Fernandes et al. [80] used a boron-doped diamond electrode (BDD) for the electrochemical oxidation of 1.7 M AO7 solution. They studied two different electrolytes (KCl and Na_2_SO_4_) that resulted in color removal with efficiencies higher than 90%. Özcan et al. [81] investigated a carbon felt cathode for AO7 degradation from an aqueous solution by electro-Fenton reaction with H_2_O_2_ and Fe^2+^ ions. They reported 92% of AO7 mineralization. They also found that the AO7 degradation rate decreased with Fe^3+^ concentrations higher than 0.1 mM. Daneshvar et al. [82] tested the electrochemical oxidation of AO7 in the presence of sulfate, chloride, and perchlorate electrolyte media at pH 3, with a mineralization rate of 75%. Zhang et al. [83] used IEO to eliminate AO7 from wastewater with a Ti/RuO_2_-Pt electrode, with a mineralization efficiency of 79.5% (from 28.5 to 5.9 mg/L) after 4 h of electrolysis. Han et al. [84] integrated electrochemical adsorption and regeneration for AO7 removal from wastewater using activated carbon fibers (ACFs). The experiment showed a regeneration efficiency higher than 70% after 10 cycles. Xia et al. [85] investigated the electrochemical oxidation of an AO7 aqueous solution (100 mg L^−1^) with a Fe-doped PbO_2_ electrode. AO7 solution decolorization and TOC were 87% and 45%, respectively, after 60 min of electrolysis (experimental conditions: 0.1 M Na_2_SO_4_, applied current density = 20 mA cm^−2^ and initial pH = 5). In the present work, AO7 removal from the water was tested using free-standing CNF electrodes, with/without Fe^2+^ ions as electro-Fenton reagent. Higher decolorization and demineralization rates were achieved in a shorter time and with lower current density. After 40 min of electrolysis at 10 mA/cm^2^ DC current and pH 3, the CNFs-Co10 electrode loaded with 25wt% of Co NPs achieved 92.5% TOC removal and complete decolorization. Moreover, the electrochemical analysis showed the high stability of the prepared electrodes in electrochemical oxidation experiments with very good AO7 solution degradation at different pH values (3 and 6).

Encapsulating Co NPs (electrocatalyst) inside CNFs to produce highly integrated electrode materials can solve the stability issue. Indeed, CNF’s excellent chemical stability could prevent Co/CoO_x_ NPs from corrosion in harsh environments. Besides the high chemical stability, Co/CoO_x_ NPs electrocatalytic activity could be further improved by the strong metal–carbon support interaction. The CNF porous nanostructures can provide fast mass transfer of the reactants and products from and to the Co/CoO_x_ NPs (catalytic active sites). The application of CNF-Co/CoO_x_ electrodes for wastewater treatment is of great interest due to their outstanding properties: (i) good cathodes for electro-Fenton oxidation; (ii) adaptability to different electro-Fenton systems (size and shape); the electrode area also can be adapted; (iii) confining Co/CoO_x_ NPs inside CNF nanovoids or channels leads to better catalytic stability because CNF-Co/CoO_x_ electrodes can be used for at least 10 cycles (40 min per cycle) without changes in their performance; and (iv) the electrode improved chemical stability greatly decreases the water treatment cost.

The degradation pathway of AO7 is widely discussed in the literature and was investigated by our group elsewhere [55]. In fact, the toxicity of the solution grows at the beginning of the electrolysis. This could be attributed to the appearance of intermediate poisonous aromatic compounds 1,2-naphthaquinone (NAPQ) and 1,4-benzoquinone (BZQ). This step is followed by a decrease in the toxicity due to the appearance of aliphatic short-chain carboxylic acids such as acetic acid and formic acid. In the end, the complete mineralization will lead to a non-toxic solution and the formation of inorganic ions, such as ammonium, nitrate, and sulphate.

## 4. Conclusions

Designing metal NPs embedded on/inside N-doped-CNFs is of great interest as electrode materials because they offer many applications for energy conversion and storage and environmental remediation. In this study, Co/CoO_x_ NPs embedded in free-standing N-doped-CNF electrodes were developed for treating wastewater containing AO7 by electro-Fenton oxidation. The composite N-doped-CNF-Co/CoO_x_ electrodes were produced by electrospinning a PAN/cobalt acetate solution, followed by thermal peroxidation in air atmosphere, and thermal carbonation in a nitrogen atmosphere. The composite N-doped-CNF-Co/CoO_x_ nanofibers possess a hierarchical structure, with the main fiber diameter of 400 nm. The free-standing N-doped-CNF-Co/CoO_x_ electrodes work as a multi-phasic electro-Fenton electrocatalyst for AO7 removal (0.1 mM initial concentration). UV-vis spectrophotometry analysis of AO7 degradation at the N-doped-CNF-Co/CoO_x_ cathode in the presence or not of an electro-Fenton reagent (Fe^2+^ ion) demonstrated the cathode’s good performance. AO7 removal with different N-doped-CNF-Co/CoO_x_ electrodes was evaluated to optimize Co/CoO_x_ NP loading and ·OH radical production in electro-Fenton oxidation. The structural and electrochemical investigations showed the strong stability of CNF-Co10 electrodes (CNFs loaded with 25 wt% Co NPs) at almost neutral pH and their very good performance for AO7 degradation at different pH values (3 and 6). After 40 min of electrolysis at 10 mA/cm^2^ DC, the N-doped-CNF-Co_10_ electrodes showed TOC removal values of 92.4% at pH 3 and 93.3% at pH 6. The enhanced performance of N-doped-CNF-Co/CoO_x_ electrodes without Fe^2+^ ions could be explained by (i) the surface-catalyzed reaction at the Co/CoO_x_ NPs surface that can allow performing the reaction at different pH values (from 3 to 6), without iron sludge precipitation, (ii) the increased H_2_O_2_ production due to the better surface area of the N-doped-CNFs-Co/CoO_x_ cathode, and (iii) the relatively good reusability of the N-doped-CNF-Co/CoO_x_ electrodes, as indicated by a decolorization rate higher than 85% of an 0.1 mM AO7 solution after 10 cycles (400 min) of degradation. Embedding metal NPs (Fe, Co, Ni, Mn) into N-doped-CNF can be considered as an effective strategy to produce electrocatalysts for the removal of organic dyes. Shortly, the CNFs electrodes will be tested for the removal of other organic matters such as antibiotics drugs (e.g., tetracycline, amoxicillin), pesticides, and microplastics from effluents to enlarge their application fields.

## Figures and Tables

**Table 1:** Characteristics of the tested cathode materials and their corresponding AO7 decolorization rate and TOC removal percentage at different pH.

Electrodes	Co Crystallite Size ^a^	R = (ID/IG) ^b^	BET Surface Area	Average Pore Diameter	Co Content ^c^	AO7 Residue ^d^	Rate Constant ^e^	TOC Removal at pH 3 ^f^	TOC Removal at pH 6 ^g^
	nm	--	cm^2^/g	nm	wt/wt%	%	min^−1^	%	%
CNF	--	1.13	7.1	13.9	No	15.8 ± 0.3	0.068	80.1 ± 0.6	81.2 ± 0.5
CNF-Fe(II) ^h^	--	1.13	7.1	13.9	No	10.5 ± 0.2	0.090	89.8 ± 0.4	--
CNF-Co3	37.0 ± 0.6	1.1	50.5	8.6	8.9 ± 0.5	10.7 ± 0.3	0.071	90.5 ± 0.3	90.5 ± 0.5
CNF-Co10	35.0 ± 0.4	1.01	115.3	3.8	25.1 ± 0.4	8.8 ± 0.3	0.080	92.4 ± 0.6	93.3 ± 0.5
CNF-Co20	32.1 ± 0.3	0.92	240.2	5.6	48.2 ± 0.7	11.1 ± 0.2	0.089	91.2 ± 0.6	91.0 ± 0.7

^a^ Crystallite size calculated from FWHM using the Scherrer equation of each diffraction profile (Figure 3). ^b^ R ratio calculated from the D and G peak relative intensities on Raman spectra (Figure 4a). ^c^ Co/CoO_x_ content (ash) in the prepared electrodes determined by TGA (Figure 5). ^d^ AO7 residue is the remaining AO7 concentration (in %) at pH 3 after 40 min measured by UV-vis spectroscopy at 480 nm. ^e^ Rate constant (K) calculated using a first-order reaction at 0–10 min (Figure 6a). ^f^ TOC removal at pH 3 and at 40 min of reaction time (Figure 6b). ^g^ TOC removal at pH 6 and 180 min of reaction time. ^h^ CNF-Fe(II) is a CNF tested with 0.1 mM AO7 and 0.20 mM Fe^2+^ as electro-Fenton reagent.

**Table 2:** Comparison of the efficiency of the self-supported CNF-Co10 electrodes and of some cathodes described in the literature for AO7 mineralization.

Cathode	Electrolyte	Initial AO7 Conc.	Current Density	Time	TOC Removal	pH	Ref.
		mM	mA·cm^−2^	min	%	--	--
rGO/carbon felt	Fe (II) + Na_2_SO_4_	0.1	30	120	73	3	[14]
Carbon felt	Fe (II) + Na_2_SO_4_	0.1	8.3	120	90	3	[78]
Stainless steel	Fe_3_O_4_ + peroxydisulfate (PDS)	0.07	8.4	90	30	3	[79]
Boron-doped diamond electrode	KCl + Na_2_SO_4_	1.7	10	600	90	3.5	[80]
CNFs-Co10	Na_2_SO_4_	0.1	10	40	92.5	3	This work

## Data Availability

Not applicable.
